# 

**Published:** 1905-09-09

**Authors:** 


**Notices of Births, Deaths, and Marriages** are inserted in this column at a charge of 2s. 6d. for an announcement not exceeding four lines.

**MARRIAGE.**

DRUITT—RADCLIFFE—August 29th, at Holy Trinity Church, Cowick, by the Rev. F., Bishop of Torquay. assisted by the Rev. C. H. Druitt, of St. Bride's, Manchester, Arthur Edward, M.R.C.S., D.P.H., second son of the late Rev. W. Druitt, of Stockbridge, Hants, to Mary Jane, second daughter of James Radcliffe, Esq. of Cowick, Yorks, and late Night Sister Soho Hospital for Women, Soho Square, W.C. (2612)

### NOTICE TO CORRESPONDENTS.

All MSS., Letters, Books for Review, and other matters intended for the Editor should be addressed THE EDITOR, "THE HOSPITAL," THE HOSPITAL BUILDINGS, 28 & 29 SOUTHAMPTON STREET, STRAND, W.C.

The Editor cannot undertake to return rejected MS., even when accompanied by stamped directed envelope.

**Notice to Advertisers and Subscribers.**

All Advertisements, Orders for Copies of THE HOSPITAL, and Business Communications should be addressed to **THE MANAGER (Not to the Editor),** THE HOSPITAL, 28 & 29 Southampton Street, Strand, London, W.C.

The Cost of Subscription, post free, is at follows:—Per week, 3½d.; per quarter, 3s. 3d.; per half-year, 6s. 6d.; per annum, 13s. Foreign and Colonial:—Per annum, 19s. 6d., payable, in all cases, in advance.

**NOTICE.—Vol. XXXVII. of "The Hospital" October 1904 to March 1905), in handsome cloth binding, will shortly be on sale at the office of this Journal, price 10s. 6d. Cases for binding the Numbers contained in this Volume 2s. Index to Vol. XXXVII., 3½d., post free.**

**The Monthly Part for September is now ready. Price 1s., by post, 1s. 3d.**

### Medical Appointments.

St. Thomas's Hospital.—The following House appointments have been made to date from Tuesday, September 5th, 1905:—Casualty Officers: **E. W. Parry**, M.R.C.S., L.R.C.P. (*Senior*); **H. A. Kisch**, M.R.C.S., L.R.C.P. (*Junior*). Resident House Physicians: **G. J. Langley**, M.B., B.S. Lond.; **C. L. Morgan**, M.B., B.S.Lond., M.R.C.S., L.R.C.P.; **H. R. Dean**, B.A., M.B., B.Ch.Oxon., M.R.C.S., L.R.C.P. (*Extension*); **A. C. Inman**, M.A., M.B., B.Ch.Oxon. (*Extension*). House Physicians to Out-Patients: **W. O. Meek**, M.R.C.S., L.R.C.P.; **F. M. Bulley**, M.A.Cantab., M.R.C.S., L.R.C.P. Resident House Surgeons: **L. E. C. Norbury**, M.B., B.S.Lond., M.R.C.S., L.R.C.P.; **D. K. Coutts**, M.B., B.S.Lond., M.R.C.S., L.R.C.P.; **J. H. Bletsoe**, M.B., B.S.Lond., M.R.C.S., L.R.C.P.; **R. J. C. Thompson**, M.R.C.S., L.R.C.P. House Surgeons to Out-Patients: **H. T. Gray**, B.A.Cantab., M.R.C.S., L.R.C.P.; **A. W. Hooker**, M.B., B.S.Lond., M.R.C.S., L.R.C.P.; **J. H. Drew**, M.B., B.S.Lond., M.R.C.S., L.R.C.P.; **H. Falk**, B.A. Cantab., M.B., B.C., M.R.C.S., L.R.C.P. Obstetric House Physicians: **E. E. Mossop**, M.B., B.S.Lond., M.R.C.S., L.R.C.P. (*Senior*); **J. M. Wyatt**, M.R.C.S., L.R.C.P. (*Junior*). Ophthalmic House Surgeons: **F. R. E. Wright**, M.B.Lond., M.R.C.S., L.R.C.P., D.P.H. (*Senior*); **C. R. B. Eyre**, M.R.C.S., L.R.C.P. (*Junior*). Special Departments: (Throat) **R. J. H. Cox**, M.R.C.S., L.R.C.P. (*Extension*); **R. H. Cotton**, M.R.C.S., L.R.C.P.; (Skin) **A. C. Birt**, M.R.C.S., L.R.C.P. (*Extension*); **W. C. A. Ward**, M.R.C.S., L.R.C.P.; (Ear) **H. S. Sington**, M.R.C.S., L.R.C.P.

### Books for

### MATRONS

**Cottage Hospital.** General, Fever, and Convalescent. Their Construction and Management. By Sir Henry Burdett. **10s. 6d.** post free.

**Furnishing and Appliances of a Cottage Hospital.** An Actual Inventory. **6d.** net; **7d.** post free.

**Hospital Expenditure:** The Commissariat. **2s. 6d.** post free.

**Cottage Hospital Case-Book of Register of Patients.** No. 1, **10s. 3d.** post free. No. 2, **12s. 10d.** post free.

**Linen Register for Large and Small Hospitals.** **7s. 6d.** net; **7s. 10d.** post free.

**Syllabus of Lectures to Nurses.** By Dr. Davidson. **1s.** post free.

**Temperature Charts.** Morning and Evening and Four Hour Charts. Size 10 in. by 8 in.

| | |
|---|---|
| 25, **1s.** | 250, **6s. 6d.** |
| 50, **1s. 6d.** | 500, **12s.** |
| 100, **2s. 9d.** | 1,000, **23s.** |

Carriage paid.

**Japanned Tin Washable Chart Holders.** Specially designed for above Charts, each, **1s.** post free, per dozen, **9s. 6d.**

### SISTERS

**Hospital Sisters and Their Duties.** By Eva E. C. Lückes, Matron London Hospital. **2s. 6d.** post free.

**Ministering Women.** The Story of the Royal National Pension Fund. **2s. 6d.** post free.

### HOSPITAL NURSES

**A Handbook for Nurses.** By Dr. J. K. Watson. **5s.** net.

**Nursing in Diseases of the Throat, Nose, and Ear.** By Macleod Yearsley. **2s. 6d.** post free.

**Elementary Treatise on the Light Treatment.** By James H. Sequeria, M.D. **2s. 6d.** net; **2s. 9d.** post free.

**Surgical Ward Work and Nursing.** By Dr. Miles. **3s. 6d.** net; **3s. 10d.** post free.

**Ophthalmic Nursing.** By Sydney Stephenson, M.B., F.R.C.S.E. **3s. 6d.** net; **3s. 10d.** post free.

**Nurses' Pronouncing Dictionary of Medical Terms and Nursing Treatment.** **2s.** post free.

### PROBATIONERS

**How to Become a Nurse: How and Where to Train.** By Sir Henry Burdett, K.C.B. **2s.** net, **2s. 4d.** post free.

**Elementary Anatomy and Surgery for Nurses.** By Dr. McAdam Eccles. **2s. 6d.** post free.

**Elementary Physiology for Nurses.** By C. F. Marshall, M.D. **2s.** post free.

**Practical Guide to Surgical Bandaging and Dressings.** By Dr. Johnson Smith. **2s.** post free.

### PRIVATE NURSES

**Art of Massage.** By Mrs. Hale. **6s.** post free.

**Pharmacy and Dispensing for Nurses.** By C. J. S. Thompson. **1s.** post free.

**Gynæcological Nursing.** By Dr. Hawkins Ambler. **1s.** post free.

**Modern Nursing in Private Practice.** By Sir William Bennett. **6d.** net, **7d.** post free.

**Pocket Book of Temperature Charts.** Containing 17 Morning and Evening and 8 Four Hour Charts. **6d.** net, **7d.** post free.

**Examination of the Urine.** By J. K. Watson, M.D. **1s.** net, **1s. 1d.** post free.

**Simple Sanitation.** The Practical Application of the Laws of Health to Small Dwellings. By Miss M. Loane. **1s.** net, **1s. 2d.** post free.

**Modern Nursing of Consumption.** By Dr. Jane Walker. **1s.** net, **1s. 2d.** post free.

**Case-Book for Private Nursing.** Size of an Exercise Book. 48 pp. **3d.** net, **4½d.** post free.

**The Scientific Press, Ltd.**

**Publishers of Nursing Text-Books, Charts, and Case-Books, of all descriptions.**

**28 & 29 SOUTHAMPTON STREET, STRAND, LONDON, W.C.**

